# Structural Remodeling of TCR–HLA-DQ8 Recognition by a β-Cell Stress-Associated C19S Insulin Neoepitope in Type 1 Diabetes

**DOI:** 10.3390/ijms27156556

**Published:** 2026-07-23

**Authors:** Rahul Mittal, Farhad Alipour, Prem Chapagain, Khemraj Hirani

**Affiliations:** 1 Diabetes Research Institute, University of Miami Miller School of Medicine, Miami, FL 33136, USA; 2 Division of Endocrinology, Diabetes, and Metabolism, Department of Medicine, University of Miami Miller School of Medicine, Miami, FL 33136, USA; 3 Department of Physics, Florida International University, Miami, FL 33199, USA; chapagap@fiu.edu; 4 Biomolecular Sciences Institute, Florida International University, Miami, FL 33199, USA

**Keywords:** type 1 diabetes, neoepitope, β-cell stress, HLA-DQ8, T cell receptor recognition, autoreactive CD4+ T cells, molecular dynamics simulation, peptide-HLA interface, insulin autoimmunity

## Abstract

Inflammatory and oxidative stress within the pancreatic islet microenvironment can alter insulin-derived peptides and generate neoepitopes that may reshape autoreactive T cell recognition in type 1 diabetes (T1D). One such modification, C19S, represents a cysteine-to-serine substitution at position 19 of the insulin B-chain and has recently been identified among human leukocyte antigen class II (HLA-II)-associated insulin neoepitopes recognized by autoreactive CD4+ T cells. Although the biological relevance of C19S has been determined, the molecular features that may distinguish C19S-specific T cell receptor (TCR) engagement from native insulin recognition remain incompletely defined. Here, we used comparative protein–protein docking, molecular dynamics (MD) simulations, interface-contact analysis, conformational landscape analysis, and binding-energy calculations to examine TCR engagement of human leukocyte antigen DQ8 (HLA-DQ8) presenting either native insulin peptide or the corresponding C19S insulin peptide. Initial modeling indicated that both peptide-HLA-DQ8 complexes were compatible with TCR-bound ternary complex formation. However, the C19S-containing complex was predicted to exhibit altered peptide-centered dynamics, changes in peptide backbone presentation, and reorganization of both TCR-peptide and TCR-HLA-DQ8 contacts. Comparative molecular mechanics Poisson–Boltzmann surface area (MM/PBSA) and molecular mechanics generalized Born surface area (MM/GBSA) analyses further suggested a distinct calculated energetic profile under the applied modeling conditions for the C19S-containing complex, with residue-level decomposition localizing energetic differences to selected interface hotspots. Together, these findings provide a molecular framework for generating hypotheses about how C19S may reshape the HLA-DQ8-presented insulin recognition surface, with implications for future experimental studies of autoreactive CD4+ T cell recognition and antigen-specific tolerogenic strategies in T1D.

## 1. Introduction

Type 1 diabetes (T1D) is an autoimmune disorder in which autoreactive T cells target β-cell-derived antigens presented by disease-associated HLA molecules, contributing to immune-mediated destruction of insulin-producing pancreatic β-cells [1,2,3,4,5,6]. Among HLA class II molecules, HLA-DQ8 is strongly associated with T1D susceptibility and contributes to autoreactive antigen recognition [7,8,9,10,11]. Structural studies of insulin peptide presentation by HLA-DQ8 have provided important insights into disease susceptibility and antigen recognition [12]. Although native insulin is a central autoantigen in T1D, the structural determinants that govern persistence of selected insulin peptides on HLA-DQ8 and their recognition by autoreactive TCRs remain incompletely defined [7,8,13]. Antigen recognition in this context is influenced by peptide sequence, binding register, peptide stability within the HLA-DQ8 groove, and the molecular surface formed by the peptide-HLA complex [14,15,16]. Even limited variation within an insulin peptide can therefore alter the structural and energetic features of the antigenic surface presented to CD4+ T cells [17,18].

Recent immunopeptidomic and functional studies have identified a cysteine-to-serine transformation at position 19 of the insulin B-chain, termed C19S, as a microenvironment-associated insulin neoepitope in autoimmune diabetes [19]. C19S insulin peptides, including InsB9-23 (C19S), have been detected in HLA-II peptidomes and are generated under conditions associated with oxidative stress, inflammatory cytokine exposure, and β-cell dysfunction. This study showed that C19S insulin peptides are present in human HLA-DQ and HLA-DR peptidomes, occur in mouse MHC-II-associated and soluble peptidomes, and are linked to stressed pancreatic islets and cytokine-activated antigen-presenting cells [19]. C19S was further associated with altered insulin autoreactivity at the single-residue level and with recognition by register-specific CD4+ T cells, including HLA-DQ8-restricted responses in the human disease context. These findings place C19S within a disease-relevant pathway in which local tissue stress can generate insulin-derived antigenic species with altered immune recognition properties.

Our previous studies examined the structural consequences of C19S at the peptide-HLA-DQ8 level [20]. Molecular dynamics (MD) simulations comparing HLA-DQ8 bound to native insulin peptide or the corresponding C19S insulin peptide indicated that the substitution may influence peptide retention within the HLA-DQ8 binding groove, hydrogen-bonding behavior, peptide mobility, and the conformational landscape sampled by the peptide-HLA-DQ8 complex [20]. These analyses provided a peptide-HLA-centered model in which C19S modifies antigen presentation by altering the stability and dynamics of the HLA-DQ8-bound insulin peptide. However, the peptide-HLA-DQ8 complex represents only one component of CD4+ T cell recognition. The antigenic surface ultimately sampled by the TCR is defined by the combined peptide-HLA-DQ8 interface and its conformational properties.

TCR engagement depends on the spatial organization, dynamics, and energetic complementarity of the peptide-HLA surface [21,22,23,24]. A cysteine-to-serine transformation within the insulin B-chain may therefore influence recognition through direct changes in side-chain chemistry and through indirect effects on peptide backbone presentation, local flexibility, HLA-DQ8 contacts, polar interaction networks, electrostatic interactions, and energetic contributions across the TCR-pHLA interface. Such effects may arise even when the peptide remains stably associated with HLA-DQ8, as TCR recognition is sensitive to distributed changes across the composite antigenic surface. Analysis of the complete TCR-HLA-DQ8-insulin peptide complex is therefore required to define how C19S-associated changes in peptide presentation may extend into the receptor-facing interface.

In the present study, we extended our previous peptide-HLA-DQ8 analysis to the full TCR-HLA-DQ8-insulin peptide ternary complex [20]. Comparative docking, MD simulations, interface-contact analysis, conformational landscape analysis, and binding-energy calculations were applied to HLA-DQ8 presenting either the native insulin peptide or the C19S insulin peptide. The analysis assessed the structural compatibility of the C19S-containing complex with TCR engagement and evaluated whether the C19S substitution was associated with differences in peptide presentation, interface organization, conformational sampling, or energetic architecture relative to the native insulin peptide complex. The workflow progressed from overall docking geometry and trajectory behavior to peptide-centered dynamics, TCR-peptide and TCR-HLA-DQ8 contacts, hydrogen-bond and salt-bridge persistence, late-phase conformational organization, and residue-level energetic decomposition. Matched computational procedures were applied to both systems to enable direct comparison under equivalent modeling conditions.

This study provides a computational structural and energetic framework for evaluating hypotheses about how a microenvironment-derived C19S insulin neoepitope may modify the TCR-facing HLA-DQ8 recognition surface. The analysis is intended to complement existing immunological findings by defining modeled molecular features that could contribute to C19S-associated recognition rather than directly measuring TCR affinity or cellular activation. Within this scope, the study addresses the interface-level organization linking insulin neoepitope formation to potential changes in autoreactive CD4+ T cell recognition. These computational observations generate testable hypotheses for future biochemical, structural, and functional studies of C19S-specific TCR recognition in autoimmune diabetes.

## 2. Results

### 2.1. C19S Preserves TCR-Compatible HLA-DQ8 Presentation Relative to WT Insulin Peptide

**Figure 1:**
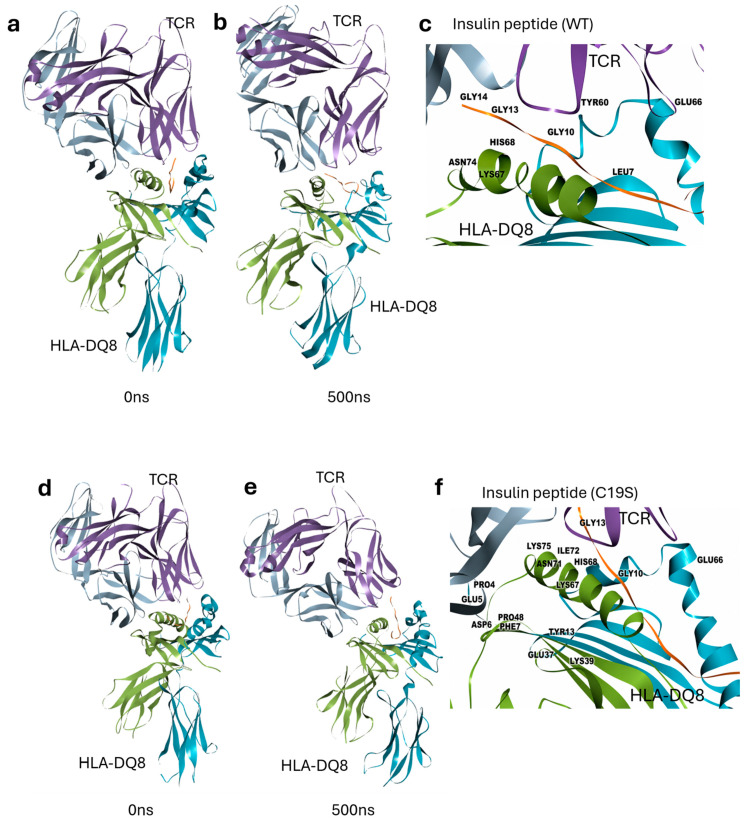
Structural models of TCR engagement with HLA-DQ8 presenting wild-type (WT) or C19S insulin peptide. Representative structural models of the TCR–HLA-DQ8–insulin peptide ternary complexes generated by protein–protein docking and refined by MD simulation. (**a**) WT initial docked conformation at 0 ns, showing the TCR positioned above the HLA-DQ8 peptide-binding groove and oriented toward the presented WT insulin peptide. (**b**) WT representative conformation after 500 ns MD simulation, demonstrating preservation of the overall TCR–HLA-DQ8–WT peptide ternary architecture. (**c**) WT magnified view of the TCR-facing HLA-DQ8 binding groove, showing retention of the WT insulin peptide beneath the TCR interface. (**d**) C19S initial docked conformation at 0 ns, showing the TCR positioned over the HLA-DQ8 peptide-binding cleft with the C19S peptide retained in the groove. (**e**) C19S representative conformation after 500 ns MD simulation, indicating maintenance of the global TCR–HLA-DQ8–C19S peptide architecture. (**f**) C19S magnified view of the HLA-DQ8 groove and receptor-facing peptide region, showing that the C19S peptide remains presented beneath the TCR-recognition surface.

Protein–protein docking was performed to model TCR recognition of HLA-DQ8 presenting either the WT insulin peptide or the C19S insulin peptide. In the WT system, the ternary complex placed the TCR over the HLA-DQ8 peptide-binding groove, with the insulin peptide positioned within the receptor-facing peptide-HLA surface. Before MD simulation, the WT peptide remained seated in the HLA-DQ8 groove while the TCR contacted the peptide–HLA-DQ8 platform in an orientation compatible with peptide MHC recognition (Figure 1a). After MD simulation, the overall ternary architecture was retained, and the TCR remained associated with the HLA-DQ8–WT peptide complex (Figure 1b). Interface-focused visualization showed that the WT insulin peptide remained presented beneath the TCR-recognition surface (Figure 1c). The C19S system showed comparable overall organization. Before MD simulation, the C19S peptide was retained within the HLA-DQ8 groove and oriented toward the receptor-contact region (Figure 1d). After MD simulation, the TCR–HLA-DQ8–C19S peptide arrangement was maintained (Figure 1e), and close-up visualization showed that the modified peptide remained positioned within the HLA-DQ8 groove near the TCR-recognition surface (Figure 1f). These modeling results suggest that the Cys-to-Ser substitution is accommodated within the modeled ternary complex without disrupting the global TCR–HLA-DQ8–peptide architecture, supporting subsequent comparison of WT and C19S modeled ternary-complex states.

### 2.2. C19S Is Associated with a More Favorable Calculated Binding Free-Energy Profile

**Figure 2:**
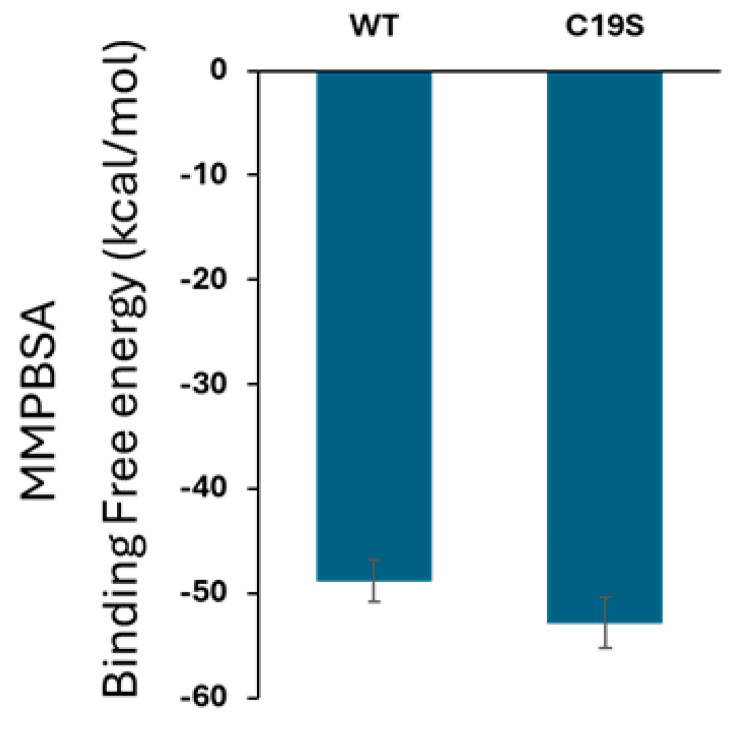
Comparative MM/PBSA binding free-energy analysis of TCR binding to WT and C19S HLA-DQ8 peptide complexes. MM/PBSA-derived binding free-energy estimates for TCR interaction with HLA-DQ8 presenting either the WT or C19S insulin peptide. The C19S-containing ternary complex exhibited a more favorable calculated binding free-energy estimate relative to the WT complex, indicating that the C19S substitution alters the predicted thermodynamic profile of TCR engagement without disrupting peptide presentation by HLA-DQ8. Error bar indicates data ± SEM.

MM/PBSA analysis was performed to compare the relative binding energetics of TCR interaction with HLA-DQ8 presenting either the WT or C19S insulin peptide. Binding free-energy estimates were calculated from sampled frames across the MD trajectories, allowing the two modeled ternary complexes to be compared under the same computational framework. In this comparison, the C19S-containing complex showed a more favorable calculated binding-energy profile than the WT complex (Figure 2). As CD4+ T cells in T1D recognize insulin-derived antigens only after they are displayed by HLA-DQ8, this energetic difference suggests that the C19S-modified peptide may change the calculated energetic features of the modeled TCR-recognition interface without preventing antigen presentation. Thus, the C19S substitution may help generate a neoepitope-like recognition surface by maintaining a TCR-bound HLA-DQ8 complex while shifting the calculated binding energetics relative to native insulin.

**Figure 3:**
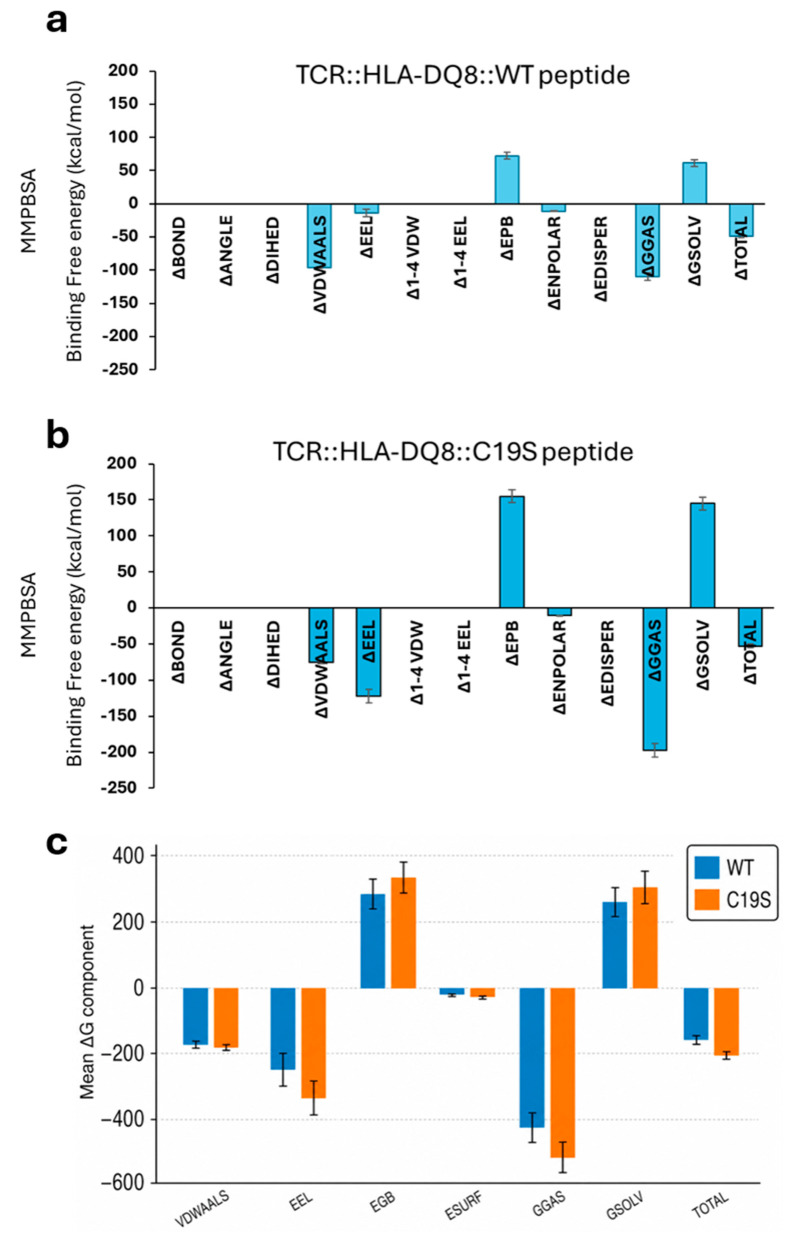
Decomposition of MM/PBSA and MM/GBSA energetic components for WT and C19S TCR–HLA-DQ8–insulin peptide complexes. Component-level MM/PBSA analysis of energetic terms contributing to calculated TCR binding in the WT and C19S ternary complexes. (**a**) Energetic component profile for the TCR–HLA-DQ8–WT insulin peptide complex. Error bars indicate mean ± SEM. (**b**) Energetic component profile for the TCR–HLA-DQ8–C19S insulin peptide complex. Molecular mechanics and solvation-energy terms were evaluated under matched conditions. Differences in the balance of van der Waals, electrostatic, polar solvation, and nonpolar solvation contributions indicate that C19S redistributes energetic contributions across the modeled TCR–HLA-DQ8–insulin peptide interface. Error bars indicate mean ± SEM (**c**) Mean MM/GBSA energetic component comparison for WT and C19S systems. Error bars indicate mean ± SD.

Component-level MM/PBSA analysis was used to examine the energetic terms contributing to the total binding free-energy estimates for the WT and C19S systems. In the WT complex, the calculated interaction profile reflected a specific balance of molecular mechanics and solvation contributions at the TCR–HLA-DQ8–peptide interface (Figure 3a). In the C19S complex, the component-energy pattern differed, indicating that the Cys-to-Ser substitution is associated with redistribution of the energetic terms contributing to the modeled TCR-bound state (Figure 3b). In the context of T1D, where autoreactive CD4+ T cells recognize insulin-derived peptides presented by HLA-DQ8, this result indicates that the C19S substitution may preserve a TCR-compatible presentation state while altering the calculated energetic organization of the recognition interface. Complementary MM/GBSA component-level analysis indicated that the difference reflected changes in the balance of energetic terms rather than a uniform shift across all components (Figure 3c).

Production-window MM/GBSA analysis separated the WT and C19S total binding-energy profiles, with the C19S complex occupying a more favorable calculated energetic range under the same analysis conditions (Supplementary Figure S1). Residue-level decomposition further localized the energetic differences to a defined set of interface residues, consistent with a reorganized hotspot pattern in the C19S complex (Supplementary Figure S2). Together, these MM/PBSA and MM/GBSA energetic analyses are consistent with the structural and contact-based findings that C19S is predicted to occupy an altered modeled TCR–HLA-DQ8 interface state.

### 2.3. C19S Alters Peptide-Centered Dynamics Within the TCR–HLA-DQ8 Complex

**Figure 4:**
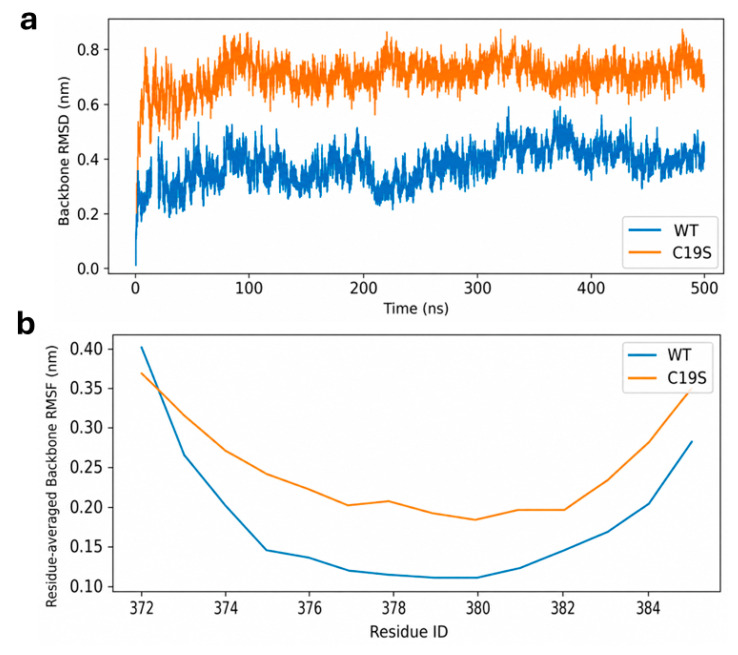
Global and peptide-residue-resolved conformational dynamics of WT and C19S TCR-HLA-DQ8-insulin peptide ternary complexes. Comparative analysis of conformational dynamics in WT and C19S TCR–HLA-DQ8–insulin peptide complexes during 500 ns MD simulation. (**a**) Backbone RMSD profiles of the complete ternary complexes, showing distinct trajectory behavior between WT and C19S systems. (**b**) Peptide-residue-resolved backbone RMSF analysis, identifying the insulin peptide as the principal region of altered flexibility.

We next evaluated whether the C19S substitution altered the dynamic behavior of the ternary complex. Backbone RMSD analysis showed distinct trajectory behavior for the C19S complex compared with the WT complex, suggesting that the C19S complex sampled a different overall conformational profile during simulation (Figure 4a). Residue-level RMSF analysis further indicated that this difference reflected a non-uniform redistribution of flexibility across the complex rather than a uniform increase in motion (Supplementary Figure S3). When analyzed by molecular component, the most evident perturbation was centered on the insulin peptide (Figure 4b), with additional changes extending into receptor-facing regions of the TCR–HLA-DQ8 assembly (Supplementary Figure S3). Consistent with this peptide-centered effect, comparison of representative late-phase peptide conformers indicated differences in backbone presentation across multiple peptide positions (Supplementary Figure S4). These analyses suggest that C19S alters the conformational behavior of the presented peptide and its local recognition environment within the modeled ternary complex.

### 2.4. C19S Is Associated with Changes in the TCR-Facing Contact Architecture

**Figure 5:**
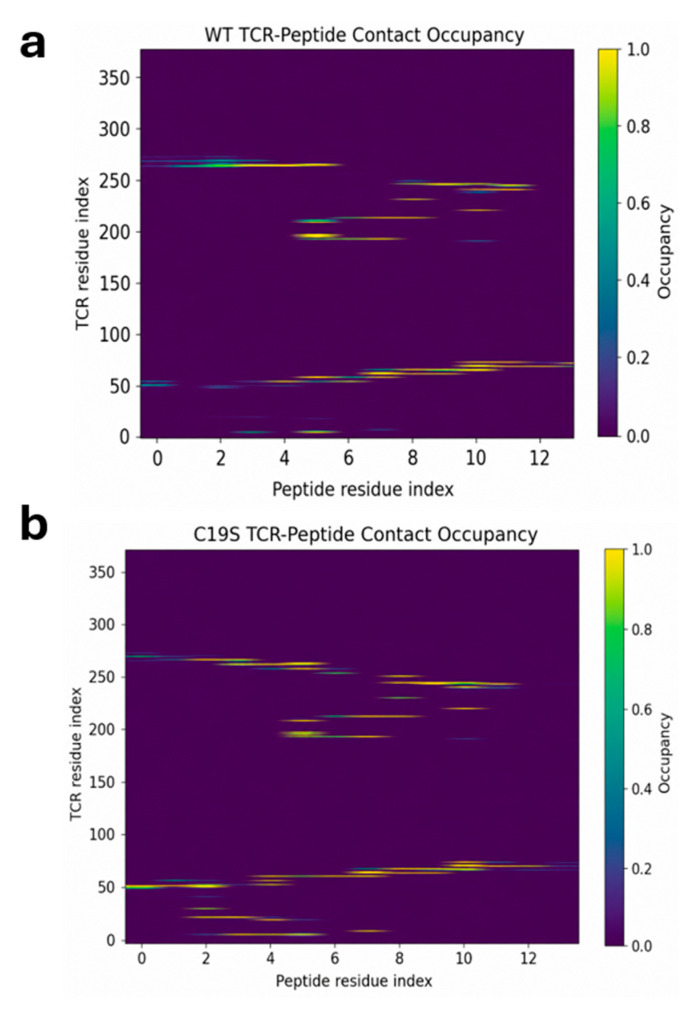
TCR-peptide contact networks in WT and C19S ternary complexes. Trajectory-based contact-occupancy analysis comparing TCR–peptide interfacial contacts in WT and C19S ternary complexes. (**a**) TCR–peptide contact occupancy map for the WT complex. (**b**) TCR–peptide contact occupancy map for the C19S complex. Contacts were defined and quantified using identical criteria across both trajectories.

To determine whether the observed dynamic differences were accompanied by altered receptor contacts, we compared contact-occupancy maps for the WT and C19S complexes. The TCR–peptide interface differed substantially between the two systems, with the C19S complex adopting a distinct pattern of peptide-facing contacts relative to WT (Figure 5a,b). This suggests that, in the modeled trajectory, the substituted peptide is engaged through a different local contact network rather than simply preserving the WT peptide-contact arrangement. TCR–HLA contact maps also differed between the two systems, suggesting that the effect of C19S extends beyond direct peptide contacts and is associated with changes across the broader receptor-facing HLA-DQ8 surface (Supplementary Figure S5a,b). Thus, the C19S substitution is associated with a coordinated reorganization of the modeled TCR–HLA-DQ8–insulin peptide interface.

### 2.5. C19S Is Associated with a Predicted Redistribution of Polar and Electrostatic Interactions

**Figure 6:**
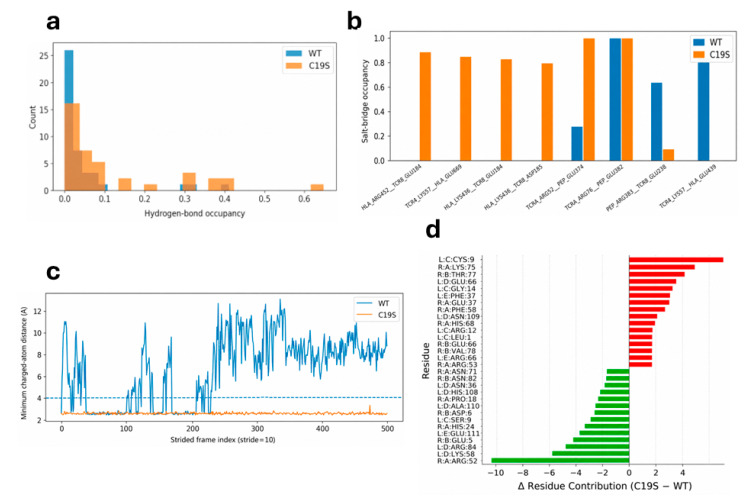
Polar and electrostatic interactions at the TCR-facing interface of WT and C19S ternary complexes. Comparative analysis of hydrogen-bonding, salt-bridge formation, diagnostic interatomic distances, and residue-level energetic contributions in WT and C19S ternary complexes. (**a**) TCR–peptide hydrogen-bond occupancy distribution in WT and C19S complexes. (**b**) Salt-bridge persistence analysis for selected interfacial charged residue pairs. (**c**) Time-dependent distance trace for the C19S-enriched TCR chain A Arg52–peptide Glu374 salt-bridge interaction, illustrating persistence of this electrostatic contact during the analyzed trajectory window. (**d**) Differential residue-level energetic contribution analysis showing residues with more favorable or less favorable calculated energetic contributions in the C19S complex relative to the WT complex.

We next examined whether the altered contact architecture was accompanied by changes in polar and charged interactions. TCR–peptide hydrogen-bond analysis identified a redistributed hydrogen-bonding pattern in the C19S complex relative to WT (Figure 6a). Salt-bridge analysis further distinguished the modeled C19S interface by identifying a remodeled set of electrostatic interactions (Figure 6b). Among these, the TCR chain A Arg52–peptide Glu374 interaction was prominent in the C19S trajectory and was observed across the analyzed window (Figure 6c). Residue-level energetic decomposition independently highlighted the same interface region among the residues contributing differently between WT and C19S (Figure 6d). The agreement between hydrogen-bonding, salt-bridge, distance-trace, and energetic-decomposition analyses supports the presence of a candidate local rearrangement of the TCR-facing interface in the C19S complex.

### 2.6. C19S Is Associated with Late-Phase Conformational Separation from WT

**Figure 7:**
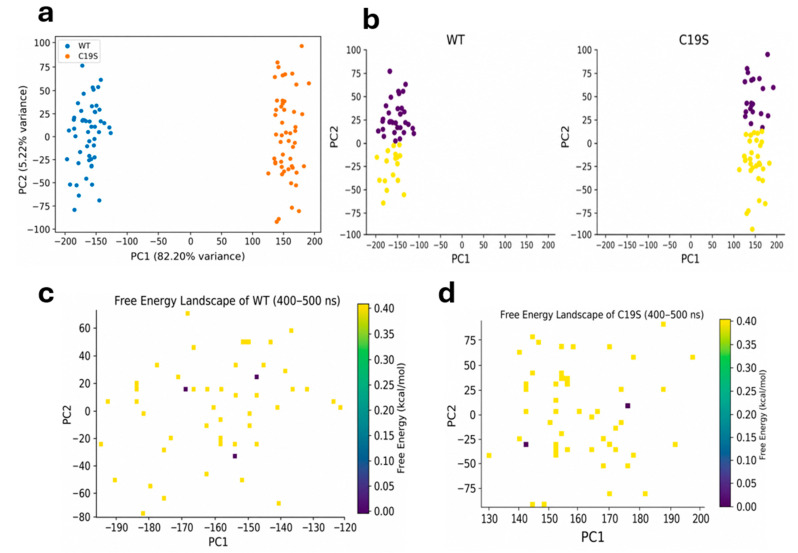
Late-phase conformational landscape of WT and C19S TCR-HLA-DQ8-insulin peptide complexes. PCA, clustering, and free-energy landscape analyses of late-phase conformational ensembles sampled by WT and C19S ternary complexes. (**a**) Joint PCA projection of WT and C19S conformations in principal component 1–principal component 2 space. (**b**) System-specific cluster occupancy analysis in principal component space. (**c**) Free-energy landscape of the WT late-phase ensemble projected onto principal component 1 and principal component 2. (**d**) Free-energy landscape of the C19S late-phase ensemble projected onto principal component 1 and principal component 2.

Given the observed changes in peptide presentation and interface contacts, we next analyzed whether the two systems occupied overlapping or distinct conformational ensembles during the late phase of simulation. Joint principal component analysis separated the WT and C19S ensembles along the dominant conformational coordinate (Figure 7a). Clustering in PCA space further indicated that the two complexes populated different regions of conformational space (Figure 7b). Free-energy landscape analysis provided a complementary view, with WT and C19S occupying distinct projected low-energy basins within the analyzed PCA space (Figure 7c,d). These analyses indicate that the C19S-containing complex samples a conformational ensemble distinguishable from the WT complex during the late simulation window.

## 3. Discussion

Neoepitope formation has emerged as an important mechanism linking β-cell stress, altered antigen presentation, and failure of immune tolerance in T1D [5,6,25,26,27]. Native insulin remains a central autoantigen in autoimmune diabetes, but recent studies indicate that the antigenic repertoire presented by disease-associated HLA-II molecules can include modified or variant insulin-derived peptides generated under inflammatory or oxidative conditions [2,4,17,28,29,30]. This concept is particularly relevant for HLA-DQ8, a major T1D-associated HLA-II molecule that presents insulin-derived peptides to autoreactive CD4+ T cells [31,32,33,34]. The identification of C19S insulin peptides in human and murine HLA-II peptidomes, together with their recognition by autoreactive CD4+ T cells, implicates this insulin B-chain modification in a disease-relevant antigen-presentation pathway [19]. The present study provides a computational structural framework by modeling the TCR-accessible HLA-DQ8 recognition surface generated following presentation of the C19S-modified insulin peptide.

The results support a model in which C19S is associated with coordinated remodeling across the TCR-HLA-DQ8-insulin peptide interface. This interpretation is consistent with studies showing that MHC-II antigenicity is shaped not only by peptide sequence, but also by peptide-binding register, peptide-MHC stability, HLA-DM editing, peptide-induced MHC-II conformational dynamics, and the geometry of the TCR-accessible recognition surface [35,36,37,38,39]. In insulin autoimmunity, this principle is particularly relevant because insulin B-chain peptides can be presented in alternative binding registers, and low-affinity or conformationally atypical peptide-HLA states may still support autoreactive CD4+ T cell responses [40,41,42]. The present computational analysis suggests that the C19S-containing complex retains compatibility with TCR engagement while modifying peptide display, receptor-facing contacts, and interface energetics. This pattern supports the view that C19S may influence structural features relevant to autoreactive recognition through distributed structural changes across the composite peptide-HLA-DQ8-TCR surface.

Our previous peptide-HLA-DQ8 simulations suggested that C19S may alter peptide retention, hydrogen-bonding behavior, peptide mobility, and the conformational landscape of the HLA-DQ8-bound insulin peptide [20]. The present ternary-complex analysis extends that work by incorporating the receptor-facing interface. This step is important as peptide-HLA-DQ8 stability is necessary for antigen presentation but does not fully define CD4+ T cell recognition. The TCR engages a composite molecular surface shaped by the peptide and surrounding HLA-DQ8 residues, and local peptide changes can influence both direct peptide contacts and HLA-mediated receptor contacts. The present findings therefore support a proposed sequential structural model in which C19S may first modify peptide accommodation within HLA-DQ8 and then contribute to an altered modeled recognition surface available to TCR.

The contact analyses provide a possible mechanistic explanation for this model. The C19S-containing complex was associated with reorganization of both TCR-peptide and TCR-HLA-DQ8 contacts, indicating that the variation affects more than the immediate peptide side-chain environment. Structural and biophysical studies of MHC-II-restricted recognition have shown that TCR specificity can be influenced by peptide-exposed residues, peptide-flanking regions, and HLA residues adjacent to the peptide-binding groove [35,38,39]. In autoimmune settings, these distributed interactions may be especially relevant as autoreactive TCRs can recognize self-peptide-HLA complexes with noncanonical binding features or altered energetic balance [40,41]. The results suggest that C19S may alter the antigenic surface through coordinated remodeling of peptide-facing and HLA-facing receptor contacts.

The C19S substitution may influence the local interaction environment of the TCR–HLA-DQ8 interface without requiring a major rearrangement of the ternary complex. As serine is more polar than cysteine, this substitution could alter the hydrogen-bonding potential at the peptide surface, redistribute local electrostatic interactions, and subtly modify local interface packing while maintaining the overall modeled binding geometry. The observed differences in contact patterns, hydrogen-bond occupancy, salt-bridge persistence, and residue-level energetic contributions suggest that the effects of C19S are distributed across neighboring peptide, HLA-DQ8, and TCR residues rather than being confined to the substituted residue alone. Consequently, altered TCR recognition may arise from modest changes in local surface complementarity, interface packing, and the organization of polar and charged interaction networks across the composite TCR-facing surface. Together, these findings provide a structural explanation for how a single peptide modification could remodel the interfacial interaction network and influence protein–protein recognition.

The polar and electrostatic interaction analyses identify a candidate local feature within the remodeled interface. The Arg52-centered interaction pattern involving the peptide region emerged across hydrogen-bond, salt-bridge, distance, and residue-level energetic analyses. This convergence does not establish experimental binding specificity, but it identifies a plausible structural element that may contribute to C19S-associated recognition. Previous studies have shown that a limited number of energetic contacts can make disproportionate contributions to peptide-MHC stability, immunodominance, and CD4+ T cell selection [35,36,43]. Within that conceptual framework, the Arg52-centered network may represent a localized interaction motif that helps distinguish the C19S-containing interface from the native insulin peptide complex. This site is therefore a potential candidate for future residue-variation studies and biophysical testing.

The conformational landscape analyses are consistent with the interpretation that C19S is associated with an altered modeled conformational state. Peptide-MHC-II complexes are dynamic assemblies, and peptide-dependent tuning of MHC-II motion has been implicated in peptide exchange, HLA-DM susceptibility, antigen persistence, and TCR engagement [35,38,39]. In the present study, the C19S-containing ternary complex occupied a conformational ensemble distinguishable from the native insulin peptide complex. This ensemble-level difference is consistent with the concept that antigenicity can be shaped by conformational selection and surface dynamics, in addition to static peptide sequence. Recent studies of cytokine-stressed human islets and HLA-associated peptidomes further suggest that inflammatory microenvironments can reshape the repertoire of antigens available for immune recognition in T1D [8,29].

The MM/PBSA and MM/GBSA energetic analyses provide a complementary but necessarily cautious interpretation. These end-point binding-energy calculations are comparative computational approaches and should not be interpreted as substitutes for direct measurements of TCR affinity, peptide-HLA stability, or kinetic lifetime. Within matched analysis conditions, however, the separation of WT and C19S energetic profiles and the redistribution of residue-level energetic contributions were consistent with the contact and electrostatic analyses. The energetic differences were concentrated in selected interface regions instead of being evenly distributed across the full complex. This pattern is compatible with a hotspot-based recognition model, in which a limited set of residues contributes substantially to interface organization and calculated stability [36,37,44]. The agreement across independent analytical layers supports the possibility that the modeled differences reflect coordinated interface remodeling rather than isolated numerical variation.

The biological relevance of this structural model is supported by the published demonstration that C19S is generated in disease-relevant microenvironments. C19S insulin peptides were reported to arise from oxidative remodeling in stressed pancreatic islets and also in cytokine-activated antigen-presenting cells, providing a potential feed-forward pathway for neoepitope formation and presentation [19]. The same study reported that C19S-specific CD4+ T cells expand at diabetes onset and acquire memory-associated features during disease progression [19]. The present study does not model the chemical generation of C19S, antigen processing, peptide competition, HLA-DM editing, or downstream T cell signaling. Instead, it focuses on the molecular state formed once the C19S peptide is presented by HLA-DQ8 and engaged by a modeled TCR. This distinction defines the scope of the study while connecting the structural analysis to a biologically established pathway.

The study also emphasizes the importance of analyzing the complete ternary complex when evaluating disease-associated insulin neoepitopes. Although peptide-HLA binding analyses provide essential insight into antigen presentation, they do not fully capture the structural determinants of CD4+ T cell activation, which depend on the TCR-accessible molecular surface and its dynamic organization within the immune synapse. Studies of TCR recognition of insulin peptides and hybrid insulin peptides have shown that changes in peptide register, peptide chemistry, and TCR docking geometry can substantially alter immune recognition in T1D [30,40,41,42]. By extending the analysis from the peptide-HLA-DQ8 complex to the complete TCR-HLA-DQ8-insulin peptide ternary complex, the present analysis provides a more comprehensive structural hypothesis for possible C19S-specific recognition mechanisms. This framework may be useful for prioritizing candidate contact residues, alternative peptide registers, and TCR-facing interface regions for experimental validation.

Several limitations should be considered. The conclusions are based on computational modeling and simulation, and the results should be interpreted as structural hypotheses. The modeled system represents one TCR-HLA-DQ8-peptide context and may not capture the full diversity of C19S-specific TCR clonotypes, HLA-II molecules, peptide registers, antigen-presenting cell states, or inflammatory conditions. The simulations do not include peptide generation, endosomal processing, HLA-DM-mediated editing, membrane localization, CD4 co-receptor engagement, or downstream signaling. End-point binding-energy calculations can also be sensitive to sampling, force-field selection, structural preparation, and dielectric assumptions. These considerations do not eliminate the internal consistency of the model, but they define the level of inference that can be drawn from the present data.

The present computational analysis was based on crystallographic structural templates, including PDB 6XCP and PDB 1JK8. Although these structures provide useful frameworks for modeling TCR engagement with HLA-DQ8-bound insulin peptides, crystallographic conditions may differ from the physiological environment. Crystal packing, buffer composition, pH, temperature, and the absence of membrane localization, CD4 co-receptor engagement, and other cellular factors may influence local conformations, side-chain orientations, peptide positioning, and TCR–HLA-DQ8 contact geometry. Although MD refinement was performed in explicit solvent under near-physiological ionic strength, the simulations remain dependent on the initial structural templates and may not capture the full conformational ensemble accessible under physiological conditions.

Future studies should evaluate the structural predictions generated by this analysis. High-resolution structures of TCR-HLA-DQ8 complexes presenting native insulin and C19S insulin peptides would allow direct evaluation of peptide backbone display, receptor-facing HLA-DQ8 contacts, and the Arg52-centered interaction network. Biophysical measurements using purified TCR and peptide-HLA-DQ8 complexes could determine whether the modeled energetic differences correspond to altered binding kinetics, complex lifetime, or thermodynamic stability. Mutagenesis of residues implicated by the contact and energetic analyses would help define their contribution to C19S-specific recognition. Functional assays using HLA-DQ8-expressing antigen-presenting cells and C19S-reactive CD4+ T cells could then connect structural predictions to antigen sensitivity, activation threshold, cytokine production, and memory-associated phenotypes.

Overall, the present study provides a molecular modeling framework linking C19S insulin neoepitope presentation to predicted alterations in TCR-HLA-DQ8 interface architecture. The results suggest that C19S may influence structural determinants of autoreactive recognition through coordinated changes in peptide display, receptor-facing contacts, polar and electrostatic interactions, conformational sampling, and energetic hotspot distribution. These computational observations complement recent immunopeptidomic and functional evidence for C19S-specific CD4+ T cell responses in T1D and extend prior peptide-HLA-DQ8 simulations by incorporating a modeled TCR-bound recognition interface [19,20]. The resulting framework provides testable hypotheses for structural, biochemical, and functional validation of C19S-specific autoreactive recognition in autoimmune diabetes.

## 4. Materials and Methods

### 4.1. Structural Preparation and System Validation of TCR–HLA-DQ8–Insulin Peptide Complexes

Structural models were generated to compare modeled TCR engagement of HLA-DQ8 presenting either the wild-type (WT) insulin peptide or the C19S insulin peptide. The human TCR structure was extracted from the ternary complex (PDB ID: 6XCP), whereas the insulin peptide-bound HLA-DQ8 structure was obtained from PDB ID: 1JK8 [12,42]. The native insulin peptide from the 1JK8 structure was retained for the WT insulin complex, while the C19S peptide was generated by substituting cysteine at position 19 with serine using the same HLA-DQ8 structural framework. The resulting HLA-DQ8–WT insulin and HLA-DQ8–C19S insulin complexes were then used for subsequent protein–protein docking with the TCR. Before docking, water molecules and non-relevant heteroatoms were removed, hydrogen atoms were added, and the structures were examined for consistency. The C19S system contained the intended cysteine-to-serine substitution within the insulin-derived peptide, whereas the WT system retained the corresponding native insulin peptide sequence. Chain assignments, residue numbering, peptide identity, and trajectory coverage were verified before analysis to ensure consistent WT and C19S residue mapping across structural, contact, conformational, and energetic comparisons. The modeled TCR system should be interpreted as one structural modeling context and not as a direct representation of the full diversity of C19S-reactive TCR clonotypes reported experimentally.

### 4.2. Protein–Protein Docking

Protein–protein docking was performed using the LightDock framework to model the interaction between the TCR and HLA-DQ8 bound to either the WT or C19S insulin peptide [45]. LightDock is a swarm-based protein–protein docking framework that samples binding orientations through glowworm swarm optimization [45,46,47,48,49]. Docking simulations were carried out using 417 docking swarms, with 199 glowworms initialized per swarm to extensively sample the protein interaction surface. The TCR was treated as the receptor, and the HLA-DQ8–peptide complex was treated as the ligand. The docking region was selected using a restraint-based approach, with residues corresponding to the expected TCR–peptide-HLA recognition region adapted from the 6XCP reference structure. Following optimization using the Glowworm Swarm Optimization algorithm, docking solutions were clustered according to the default LightDock clustering procedure. The highest-ranked representative pose with the lowest docking score from the resulting clusters was selected for subsequent structural analysis.

### 4.3. MD Simulations and Trajectory Processing

The selected TCR–HLA-DQ8–peptide docking models were subjected to all-atom MD simulations using GROMACS [50,51,52,53]. Each ternary complex was placed in a periodic simulation box and solvated with explicit SPC water molecules. Counterions were added to neutralize the systems, and NaCl was added to approximate physiological ionic strength, with ionic strength adjusted to 0.15 M NaCl to mimic physiological conditions. Energy minimization was performed using the steepest descent algorithm until convergence. The minimized systems were equilibrated under constant volume and temperature conditions, followed by constant pressure and temperature equilibration. After equilibration, production simulations were performed for 500 ns for each system [54,55,56,57,58,59]. Long-range electrostatic interactions were treated using the Particle Mesh Ewald method, and bonds involving hydrogen atoms were constrained using the LINCS algorithm [60,61,62,63,64]. Simulation trajectories were processed using matched alignment and sampling procedures for WT and C19S complexes, and representative frames were extracted for structural visualization, RMSD analysis, and downstream comparative analyses.

### 4.4. MM/PBSA and MM/GBSA Binding-Energy Analyses

Binding-energy calculations were performed using both MM/PBSA and MM/GBSA approaches to compare the relative energetic profiles of TCR engagement with HLA-DQ8 presenting either the WT insulin peptide or the C19S insulin peptide. These analyses were performed using the gmx_MMPBSA package [65], using molecular dynamics trajectories generated by GROMACS [50,51,52,53]. MM/PBSA calculations were performed to estimate the relative binding free energy of TCR engagement across sampled trajectory frames. Representative frames were extracted from the MD trajectories and analyzed using the same computational protocol for both systems. Binding free energy was calculated as the difference between the free energy of the TCR–HLA-DQ8–peptide complex and the combined free energies of the separated receptor and ligand components. Molecular mechanics terms, including van der Waals and electrostatic contributions, were evaluated together with polar and nonpolar solvation terms. Component-level energy contributions were compared between the WT and C19S complexes to assess the possible energetic basis of the calculated binding profiles.

MM/GBSA calculations were performed using frames from the stabilized production window of the WT and C19S trajectories to evaluate binding energetics under matched sampling conditions. Total binding-energy profiles were calculated for both complexes, and individual energetic components, including van der Waals, electrostatic, polar solvation, nonpolar solvation, gas-phase, solvation, and total energy terms, were compared between systems. Residue-level MM/GBSA decomposition was then performed on the same production-window frames to identify interface residues with differential energetic contributions in the C19S complex relative to WT. Per-residue contribution profiles were compared between systems to determine whether the calculated energetic differences were broadly distributed across the interface or localized to selected interaction hotspots.

### 4.5. RMSD, RMSF, and Peptide-Presentation Geometry Analyses

Global conformational behavior was evaluated using backbone root mean square deviation (RMSD) analysis. Local flexibility was evaluated using backbone root mean square fluctuation (RMSF) analysis at the residue level. RMSF values were calculated across the complete complex and summarized by molecular component, including the TCR chains, insulin-derived peptide, and HLA-DQ8 chains. These analyses were performed using matched procedures for the WT and C19S systems to compare conformational sampling and local flexibility under equivalent analysis conditions. Representative late-phase structures were extracted from the stabilized portion of each trajectory to compare peptide presentation in the WT and C19S complexes. Peptide backbone geometry was evaluated by aligning representative WT and C19S conformers and calculating residue-wise backbone displacement across the insulin-derived peptide.

### 4.6. Interface Contact, Hydrogen-Bond, and Salt-Bridge Analyses

Interface contacts were analyzed to compare TCR–peptide and TCR–HLA-DQ8 interaction patterns between the WT and C19S complexes. Contacts were calculated across the trajectories and summarized as contact occupancies. TCR–peptide and TCR–HLA contacts were analyzed separately to distinguish direct peptide-recognition effects from broader changes across the receptor-facing HLA-DQ8 surface. Identical contact definitions and occupancy calculations were applied to both systems. Hydrogen-bond persistence was calculated across the trajectories to compare polar interaction networks in the WT and C19S complexes. TCR–peptide and TCR–HLA-DQ8 hydrogen bonds were analyzed separately. Salt-bridge analysis was performed for charged residue pairs at the TCR–peptide and TCR–HLA-DQ8 interfaces. For selected diagnostic electrostatic interactions, interatomic distance traces were calculated across the analyzed trajectory to evaluate interaction persistence over time.

### 4.7. Principal Component Analysis, Clustering, and Free-Energy Landscape Analysis

Late-phase conformational ensembles from the WT and C19S trajectories were combined and subjected to joint principal component analysis (PCA). This approach allowed both systems to be projected into a common conformational coordinate space. The projected conformations were further analyzed by clustering in principal component space to evaluate whether the WT and C19S systems populated overlapping or distinct conformational regions. Free-energy landscapes were constructed from the principal component projections to compare the low-energy conformational basins sampled by each system.

All WT and C19S comparisons were performed using matched structural alignment, trajectory sampling, contact definitions, projection methods, and energetic-analysis procedures.

## Data Availability

The original contributions presented in this study are included in the article/Supplementary Material. Further inquiries can be directed to the corresponding authors.

## Supplementary Materials

The following supporting information can be downloaded at: https://www.mdpi.com/article/10.3390/ijms27156556/s1.
