# Supplementary material for: Structural Remodeling of TCR–HLA-DQ8 Recognition by a β-Cell Stress-Associated C19S Insulin Neoepitope in Type 1 Diabetes
Source: Int J Mol Sci. 2026 Jul 23;27(15):6556. doi: 10.3390/ijms27156556 (PMC13464941; doi:10.3390/ijms27156556)
Supplement: Supplementary file 1 [file ijms-27-06556-s001.zip › Supplementary Figure Legends.pdf]

Supplementary Figure Legends

Supplementary Figure S1. Production-window MM/GBSA binding free-energy profiles of WT and C19S TCR–HLA-DQ8–insulin peptide complexes. Comparative MM/GBSA binding free-energy estimates calculated from the stabilized production phase (400–500 ns) of the molecular dynamics trajectories for TCR interaction with HLA-DQ8 presenting either the wild-type (WT) or C19S insulin peptide. Individual trajectory frames are shown together with the mean binding free energy (dashed lines) for each system.

Supplementary Figure S2. Residue-level MM/GBSA energetic decomposition of WT and C19S ternary complexes. Mean per-residue MM/GBSA energy decomposition comparing energetic contributions of interface residues to TCR binding in WT (blue) and C19S (orange) TCR–HLA-DQ8–insulin peptide ternary complexes. Negative values indicate energetically favorable contributions to binding, whereas positive values represent less favorable contributions.

Supplementary Figure S3. Residue-level backbone RMSF analysis of WT and C19S TCR–HLA-DQ8–insulin peptide complexes. Residue-level backbone RMSF profiles calculated across the complete WT and C19S ternary complexes during the 500 ns MD simulations. WT is shown in blue, and C19S is shown in orange. Residues are grouped according to molecular component, including the T cell receptor (TCR; residues 1–371), the insulin peptide (residues 372–385), and HLA-DQ8 (residues 386–812).

Supplementary Figure S4. Residue-wise comparison of WT and C19S peptide backbone conformations. Residue-wise backbone RMSD values comparing representative WT and C19S insulin peptide conformations across peptide residue IDs 372–385. The plot shows position-dependent differences in peptide backbone geometry between the two systems.

Supplementary Figure S5. C19S-associated changes in TCR–HLA-DQ8 contact networks. Trajectory-based contact-occupancy analysis comparing TCR–HLA-DQ8 interfacial contacts in WT and C19S ternary complexes. **(a)** TCR–HLA-DQ8 contact occupancy map for the WT complex. **(b)** TCR–HLA-DQ8 contact occupancy map for the C19S complex. Contacts were defined and quantified using identical criteria across both trajectories.
